# Advancing patient care with tau pathology biomarkers

**DOI:** 10.1002/alz70856_102911

**Published:** 2025-12-25

**Authors:** Howard M Fillit

**Affiliations:** ^1^ Alzheimer's Drug Discovery Foundation, New York, NY, USA

## Abstract

Stratifying patients by tau burden has emerged as a critical step in advancing Alzheimer's disease (AD) research and care. Data from clinical trials such as the LEQ and DONA studies reveal that tau pathology correlates strongly with patient outcomes, underscoring its importance in predicting disease progression. This finding is further supported by imaging and cerebrospinal fluid (CSF) studies, which highlight the role of tau in forecasting clinical trajectories and guiding therapeutic interventions.

Currently, blood‐based biomarkers focus predominantly on amyloid pathology, with tests like plasma amyloid‐β (Aβ) ratios aiding in early detection. However, these biomarkers fall short in addressing the complexity of AD, particularly the contributions of tau pathology. To truly improve patient outcomes, the development of cost‐effective and non‐invasive blood tests capable of predicting tau pathology independently of amyloid is essential. Such tools would enable a more comprehensive assessment of AD pathology and facilitate earlier, more precise interventions.

Patient stratification based on both amyloid and tau biomarkers offers clinicians a more nuanced understanding of disease biology. By tailoring therapeutic approaches to individual biomarker profiles, this strategy has the potential to optimize treatment efficacy and slow disease progression. Beyond amyloid and tau, integrating additional biomarkers that reflect the broader biology of aging will be pivotal in the evolution of precision medicine for AD.

The integration of amyloid and tau biomarkers marks the beginning of a new era in AD care, enabling more targeted interventions and fostering the development of next‐generation therapies. This paradigm shift not only aligns with the principles of precision medicine but also sets the stage for a more holistic approach to tackling the multifaceted nature of aging‐related diseases. As efforts to bring tau biomarkers to clinical practice continue, they hold the promise of transforming AD diagnosis, treatment, and research on a global scale.

